# Universal goniometer and electro-goniometer intra-examiner reliability in measuring the knee range of motion during active knee extension test in patients with chronic low back pain with short hamstring muscle

**DOI:** 10.1186/s13102-019-0116-x

**Published:** 2019-03-22

**Authors:** MohammadBagher Shamsi, Maryam Mirzaei, Seyyed Saeed Khabiri

**Affiliations:** 1 Department of Rehabilitation and Sport Medicine, School of Allied Medical Sciences, Kermanshah University of Medical Sciences, Kermanshah, Iran; 2 Department of Orthopedic Surgery, Taleghani Hospital, Faculty of Medicine, Kermanshah University of Medical Sciences, Kermanshah, Iran

**Keywords:** Universal goniometer, Electro-goniometer, Intra-examiner repeatability, Active knee extension test, Chronic low back pain, Hamstring muscle

## Abstract

**Background:**

Both universal goniometer and electro-goniometer are used for measuring joint range of motion in physiotherapy. Active knee extension test is a way to assess hamstring shortness in patients with chronic low back pain. The aim of this study was to assess universal goniometer and electro-goniometer reliability in measuring knee angle during active knee extension test.

**Methods:**

This was an intra-examiner reliability study between three measurements of knee extension angle that conducted on 45 patients with chronic low back pain having short hamstring muscle that referring to Kermanshah University of Medical Sciences clinic from 2016 to 2017. Knee extension angle was measured three times during active knee extension test with both universal goniometer and electro-goniometer.

The measurement of knee extension angle was done at the beginning, middle and the end of one single session by one experienced physiotherapist.

The intra-class correlation coefficient (ICC) and standard error of measurements (SEM) were used to quantify intra-examiner reliability.

**Results:**

For both methods, the reliability test values were found to be greater than 0.7 in the range of 0.92 to 0.99 (CI 95% ranged over = 0.94 to 0.99), which are classified as good reliability. The SEMs ranged from 1.04° to 2.16° for both scales.

**Conclusion:**

Universal goniometer in clinical evaluations of patients (as they are easy to be employed) and electro-goniometer in laboratory studies (as they are more accurate) are reliable.

## Background

All over the world, back pain imposes a large amount of direct and indirect costs to health care and treatment systems and is one of the main reasons for the absence of people from the workplace [1–3]. Over 80% of people have experienced at least one back pain during their life [4, 5]. Several studies have reported various general factors such as obesity, smoking, and long-term sedation as risk factors for back pain [6, 7]. Also, hamstring shortness is known as a risk factor for back pain [4, 5]. So that having a short hamstring muscle is usually reported in patients with back pain. In fact, it is assumed that a short hamstring can disrupt the biomechanics of the pelvis and the lumbar and lead to back pain [8, 9].

Different techniques are used for the shortened muscle stretching and increase flexibility, such as positional stretching, dynamic stretching, static stretching, and ballistic stretching which can include static stretching exercises. Although it will be possible to fix the shortened muscle with continuous and static stretch and with this technique, the structure of the soft tissues can be altered and it can cause the collagen fibers and their length increase. But these changes are not lasting [10–12].

On the other hand, evidence has shown that using long-term strengthening exercises results in a lasting and durable change in muscle [13].

Clinically, hamstring length can be measured indirectly which is carried out by measuring hip’s range of motion (ROM) during the passive straight leg raise (SLR) or active knee extension (AKE) tests. According to Lusin and Gajdosik, to measure the length of the hamstring, the AKE test is recommended which is a better choice than the passive SLR. AKE test has very high reliability [14, 15]. This test is remarked safe, as the participant dictates his/her end of range [16].

Given that the standard (universal) goniometer is affordable and available if used appropriately, it is a useful measurement tool that can be used to do the AKE test. On the other hand, using the electro- goniometer, the AKE test can be performed with high precision, but the electro- goniometer, unlike the standard goniometer, is not easily accessible and it is costly [17].

Considering the mentioned cases and the importance of hamstring and the tests for checking the length of it, the aim of this study was set out to determine the within-session reliability of universal goniometer and electro-goniometer for measuring knee extension during AKE test in a single session. Comparing these two different instruments in measuring knee angle may be useful in choosing a proper method in clinical practice and/or research projects.

## Methods

### Study design and participants

This intra-examiner reliability study of two methods was part of another parallel, double-blinded, randomized, controlled clinical trial. The above study was in accordance with the guidelines laid down in the Declaration of Helsinki, (registration no.IRCT201507258035n2 in the Iranian Registry of Clinical Trials) and undertaken in the Kermanshah University of Medical Science (with the identification No. kums.rec.1395.169 in the Research committee of Ethics at Kermanshah University of Medical Science).

Because the present work was part of another clinical trial with variables related to EMG activity of shortened hamstring muscles, the sample size was calculated based on a study conducted by Meroni et al. [9]; so, by considering a confidence interval of 95% and power of 80%, the sample size was determined to be 45 patients.

Forty-five Participants with chronic low back pain (LBP) with short hamstring muscle were selected from patients referring to Kermanshah University of Medical Sciences clinic by an experienced physiotherapist from 2016 to 2017 (by implementing the convenience sampling method). The criteria for entering the study included having LBP for more than 3 months and the informed consent of the individual and tangible shortness of hamstring muscle in the clinical examination and in conducting the SLR test. Study exclusion criteria included orthopedic and neurological disorders, history of lower extremity hamstring damage in the past year, diseases such as arthritis, ligament and meniscus damage, and history of spinal surgery. For example, if the participant had a disc herniation, he/she would be excluded from the study.

### Procedure and measuring tools

The amount of extension was measured using a universal-goniometer and both electro-goniometer. Each test was repeated three times at one single session. The measurement of knee extension angle was done at the beginning, middle and the end of the session by one experienced physiotherapist.

Measurement of knee joint extension in active AKE test using standard goniometer:

In this test, each subject was in supine position, while a small pillow was placed beneath his head and neck [18].

Then the legs and thighs of the other lower extremity were fixed to the bed with straps. The knee flexion axis was being marked by a pen and from this point, a line was being drawn to the greater trochanter of the femur and one other line to the external malleolus of the ankle. These lines were used to measure knee joint angles. The goniometer axis was placed on the knee axis and its arm was placed along the line drawn on the thigh and the other arm was placed along the line drawn on the leg. Using two wooden legs on the sides of the thigh and a horizontal bar on which they were placed, the intended hip joint in 90^0^ and the vertical position of the thigh was being maintained. The subject was asked to do the active knee extension slowly within 3 s as far as he/she could while the ankle was in a neutral position [19].

**Fig. 1:**
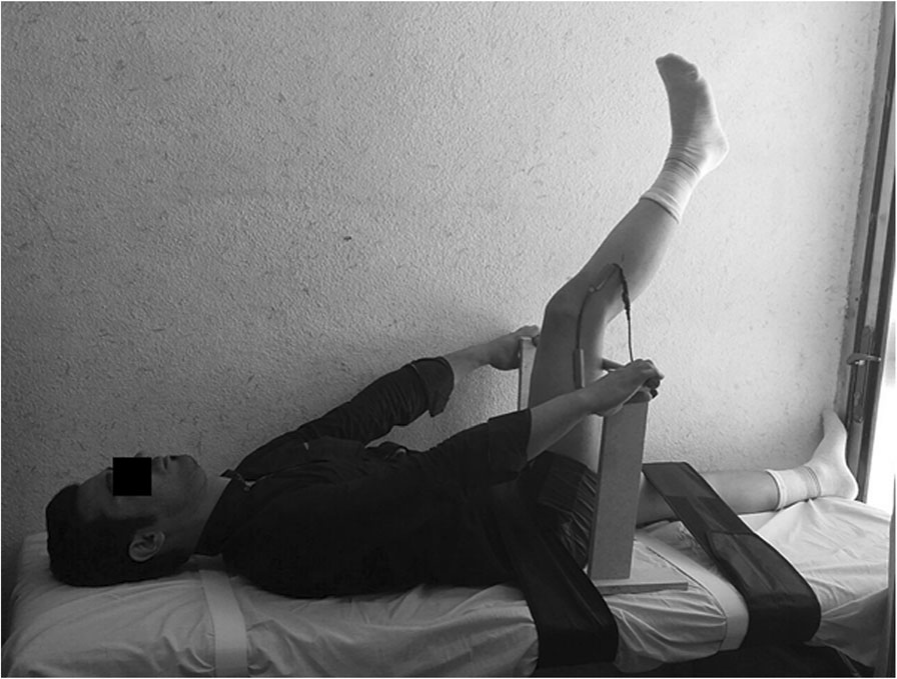
Active straight leg raise test and hip flexion angle

Then, when the active knee extension movement was completed and the subject was attempting to keep this situation for a second, the angle indicated by the goniometer was the extension angle of the knee joint [18] (Fig. 1).

Measurement of knee joint extension in AKE test using electro-goniometer:

Two electro-goniometer sensor arms were stuck on the lines which had been drawn on the legs and thighs in the previous test, by using the tape. The patient was placed in a test condition that was mentioned in the previous test and was asked to actively extend the knee as much as he/she could. The angle between the two arms was recorded by the device software and it was determined as the test result.

### Data analyses

Data were analyzed using SPSS software (version 21). Data were reported in terms of mean ± SD and frequency (percentages).

Kolmogorov-Smirnov test was used to examine the normal distribution of data. The intra-class correlation coefficient (ICC) with 95% confidence interval was used to show the intra-examiner (within-session) reliability of the measurements. Also, standard error of measurements (SEM) was used to express the absolute repeatability by the same rater. The ICC values higher than 0.90, between 0.80–0.70 and lower than 0.70 were considered as excellent, good/ moderate and poor reliability, respectively [20].

## Results

Based on the results, 31(68%) of the patients were male and 14(32%) were female. The age range was 19–59 years (Table 1). The Smirnov-Kolmogorov test showed that the research data had a normal distribution (*p* > 0.05).

**Table 1:** Baseline characteristics of the LBP patients (*n* = 45)

Variables	Mean ± SD	Min	Max
Age(year)	38.80 ± 11.14	19	59
Height (cm)	172.29 ± 11.12	151	191
Weight (kg)	79.32 ± 14.39	56	102
BMI (kg/m^2^)	26.69 ± 3.55	18	26.69

*BMI* body mass index, *LBP* low back pain

The mean values ± SD of the angles obtained by universal goniometer after three times of testing were 155.38° ± 7.39°. While this value was obtained in electro-goniometric method 144.74° ± 21.71°.

The absolute and relative repeatability of the angles measured by both instruments is shown in Table 2.

**Table 2:** Mean ± SD, reliability coefficient and SEM of knee extentison degree mesured by electro goniometer and universal goniametr (n = 45)

Scale	First assessment Mean ± SD	Second assessment Mean ± SD	Third assessment Mean ± SD	ICC (95% CI)	SEM°
Electro-goniometer	143.81 ± 22.69	146.67 ± 19.45	147.29 ± 18.94	0.99 (0.98–0.99)	2.16°
Universal goniometer	154.14 ± 7.01	155.76 ± 7.69	157.48 ± 6.83	0.96 (0.94–0.96)	1.04°

ICC (95% Confidence Interval) was reported/ ICC: Intra-class correlation coefficient/ SEM: Standard error of measurements

As shown in Table 2, in the present study, for both instruments, the relative reliability coefficients (ICC) for the three measurements using the same rater were found to be greater than 0.7 in the range of 0.92 to 0.99, which are classified as excellent repeatability. Additionally, the absolute reliability values (SEM) of the 2 instruments were estimated to be 1.04° for universal goniometer and 2.16° for electro-goniometer, respectively.

## Discussion

The purpose of this study was to investigate the intra-examiner repeatability of two methods of universal goniometer and electro-goniometer in measuring the extension angle of the knee joint during the active extension test in chronic back pain patients with hamstring muscle shortness. The reliability values obtained from the ICC test and the SEM indicated a good intra-examiner for both instruments, but this reliability was higher in electro-goniometer method.

In a similar study like many other ones the intra-tester reliability of goniometric measurements for the universal goniometer during active knee flexion measurement was high (0.997) which is in line with our results [6]. In another recent study that compared the results for the measurement of knee joint flexion angle using electro-goniometer and universal goniometer, the ICC for electro-goniometer was 0.87–0.88 and for universal goniometer was 0.78–0.82, which are somehow lower than ours but with the same order (electro-goniometer reliability more than universal goniometer) [21].

In physiotherapy, measuring the range of the joint motion is used as a tool for evaluating the patient’s physical condition [20]. To measure the range of motion of joints, the use of universal goniometers is common in physiotherapy clinics and research centers. The electro-goniometer evaluates the mechanical condition of the joint by electronic components and is used today in research centers. Although radiography is considered as the gold standard of measuring the range of joint motion, however, it is limited due to its radioactive radiation and the failure to repeat the use of it [22]. Compared to radiography, the assessment of the angle with the goniometer shows a high level of accuracy [23].

According to Norkin and White’s study [20], measuring a joint angle with a universal goniometer has moderate to excellent reliability. It can therefore, be used as a repeatable device for measuring the range of motion of the joint. Simatti [24], compared two methods of measuring the wrist pronation and supination by using a goniometer and described the inter-rater reliability of these methods as excellent. He did not evaluate the intra-rater reliability in this study. Repeatability or reliability is a measurement method, carried out by repeated measurements of a single variable on a single person or object in the same conditions [25]. Rowe in his study found that measuring the angle with an electro-goniometer is not affected by environmental factors such as heat, electrical interactions, convection flows, and sound [26]. Therefore, it is possible to use it in different conditions. Armstrong showed that in measuring the angle of flexion and extension of the elbow, the intra-rater reliability of the electro-goniometry is greater than universal goniometer (ICC 0.95 for flexion and ICC 0.89 for extension) [27]. In Singh study, he stated that in measuring the wrist rotating movements by using an electro-goniometer, the reliability of the inter-rater and the intra-rater were satisfactory (Inter-rater ICC was 0.92 and the first tester ICC was 0.92 and 0.93 for the second tester) [28]. Results of other studies showed higher reliability of intra-rater and inter-rater in the electro-goniometer (except for the reliability of the inter-rater in assessing the flexion of the wrist joint). This can be due to the fact that the electro-goniometer is fixed on the skin surface in the other joints more than the wrist [25]. In the Camassuti’s study, an excellent intra-rater and inter-rater reliability were obtained for the electro-goniometer and it was introduced as a reliable method for clinical measurements [25]. According to Norkin and White, reliability levels show higher scores whenever the same examiner performs the successive measurements [20]. In another study, Singh referred to a bit less intra-rater reliability than inter-rater reliability on all of the measurements with an electro-goniometer [28].

Despite the many electro-goniometer advantages, the use of this tool is subject to cross-talk error. Fultran reports that errors that occur by this device are in conjunction with the distortion degree that occurs in the springs of the axis of this device, especially when it is located at the end of the range of motion. Meanwhile, even if the device is fixed on the skin, the patient’s body specification such as skin flexibility, bone structure, fat, and muscle, may be effective in altering the result of the measurement [25]. Overall, the results of this study are in line with the results of similar studies and show the high reliability of universal goniometer and electro-goniometer in measuring the joint angles, but this reliability is higher in electro-goniometer. The convenience of using the universal goniometer and its availability makes it more general to use and only the higher accuracy of the electro-goniometer in measuring the joint angles makes it more justifiable in laboratory studies.

Electro-goniometer is considered as a potentially efficient method for quantifying joint angle compared against observational analysis like universal goniometer [29].

Due to the limitations and prolongation of the patient’s treatment, tests were performed three times all in 1 day and if this repetition was performed on different days it could increase the accuracy of the work (test-retest reliability). We did not evaluate the inter-rater reliability in this study. Measurements were done by only one assessor and if there was more than assessor, inter-rater reliability could be assessed.

In future studies, test-retest reliability assessment of universal goniometer and electro-goniometer with more participants and more assessors in different days is suggested.

## Conclusion

Based on the results of this study, both universal goniometer and electro-goniometer methods had good reliability, but this reliability was higher in the electro-goniometer method. It can be concluded that as the universal goniometer is easy to be employed, it can be used in clinical evaluations of patients and as the electro-goniometer is more accurate, it can be used in laboratory studies.

## Abbreviations

AKE: Active knee extension test
ICC: Interclass correlation coefficient
LBP: Low back pain
SEM: Standard error of measurements

## References

[1] Legge D (2015). Acupuncture treatment of chronic low back pain by using the Jingjin (meridian sinews) model. J Acupunct Meridian Stud.

[2] K P (2015). The use of passive straight leg raising test: a survey of clinicians. Malays Orthop J.

[3] Raftry SM, Marshall PW (2012). Does a ‘tight’hamstring predict low back pain reporting during prolonged standing?. J Electromyogr Kinesiol.

[4] Shrier I, Ehrmann-Feldman D, Rossignol M, Abenhaim L (2001). Risk factors for development of lower limb pain in adolescents. J Rheumatol.

[5] Kennedy C, Kassab O, Gilkey D, Linnel S, Morris D (2008). Psychosocial factors and low back pain among college students. J Am Coll Heal.

[6] Wong AY, Karppinen J, Samartzis D (2017). Low back pain in older adults: risk factors, management options and future directions. Scoliosis Spinal Disord.

[7] Shemory ST, Pfefferle KJ, Gradisar IM (2016). Modifiable risk factors in patients with low back pain. Orthopedics.

[8] MassoudArab A, RezaNourbakhsh M, Mohammadifar A (2011). The relationship between hamstring length and gluteal muscle strength in individuals with sacroiliac joint dysfunction. J Man Manip Ther.

[9] Meroni R, Cerri CG, Lanzarini C, Barindelli G, Della Morte G, Gessaga V, Cesana GC, De Vito G (2010). Comparison of active stretching technique and static stretching technique on hamstring flexibility. Clin J Sport Med.

[10] Morse CI, Degens H, Seynnes OR, Maganaris CN, Jones DA (2008). The acute effect of stretching on the passive stiffness of the human gastrocnemius muscle tendon unit. J Physiol.

[11] Muragod AR, Pathania T (2017). Effects of static stretching and neurodynamic mobilization on hamstring flexibility in elderly population-a randomized clinical trial. IJAR.

[12] Sairyo K, Kawamura T, Mase Y, Hada Y, Sakai T, Hasebe K, Dezawa A (2013). Jack-knife stretching promotes flexibility of tight hamstrings after 4 weeks: a pilot study. Eur J Orthop Surg Traumatol.

[13] Aquino CF, Fonseca ST, Gonçalves GG, Silva PL, Ocarino JM, Mancini MC (2010). Stretching versus strength training in lengthened position in subjects with tight hamstring muscles: a randomized controlled trial. Man Ther.

[14] De Weijer VC, Gorniak GC, Shamus E (2003). The effect of static stretch and warm-up exercise on hamstring length over the course of 24 hours. J Orthop Sports Phys Ther.

[15] Gajdosik R, Lusin G (1983). Hamstring muscle tightness: reliability of an active-knee-extension test. Phys Ther.

[16] Shepherd E, Winter S, Gordon S (2017). Comparing hamstring muscle length measurements of the traditional active knee extension test and a functional hamstring flexibility test. Physiother Rehabil.

[17] Radwan A, Bigney KA, Buonomo HN, Jarmak MW, Moats SM, Ross JK, Tatarevic E, Tomko MA (2015). Evaluation of intra-subject difference in hamstring flexibility in patients with low back pain: an exploratory study. J Back Musculoskelet Rehabil.

[18] Webright WG, Randolph BJ, Perrin DH (1997). Comparison of nonballistic active knee extension in neural slump position and static stretch techniques on hamstring flexibility. J Orthop Sports Phys Ther.

[19] Nelson RT, Bandy WD (2004). Eccentric training and static stretching improve hamstring flexibility of high school males. J Athl Train.

[20] Norkin CC, White DJ. Measurement of joint motion: a guide to goniometry: FA Davis; 2016.

[21] Lim CC, Affandi M, Basah SN, Din MY (2018). Evaluating lower limb joint flexion by computerized visual tracking system and compared with Electrogoniometer and universal goniometer. J Telecomm Electronic Comp Eng.

[22] Cleffken B, van Breukelen G, van Mameren H, Brink P, Damink SO (2007). Test-retest reproducibility of elbow goniometric measurements in a rigid double-blinded protocol: intervals for distinguishing between measurement error and clinical change. J Shoulder Elb Surg.

[23] McVeigh KH, Murray PM, Heckman MG, Rawal B, Peterson JJ (2016). Accuracy and validity of goniometer and visual assessments of angular joint positions of the hand and wrist. J Hand Surg Am.

[24] Cimatti B, Marcolino AM, Barbosa RI, Cássia Registro D, Fonseca M (2013). A study to compare two goniometric methods for measuring active pronation and supination range of motion. Hand Therapy.

[25] da Silva Camassuti PA, Marcolino AM, Tamanini G, Barbosa RI, Barbosa AM, de Cássia Registro Fonseca M (2015). Inter-rater, intra-rater and inter-instrument reliability of an electrogoniometer to measure wrist range of motion. Hand Therapy.

[26] Rowe P, Myles C, Hillmann S, Hazlewood M (2001). Validation of flexible electrogoniometry as a measure of joint kinematics. Physiotherapy.

[27] Armstrong AD, MacDermid JC, Chinchalkar S, Stevens RS, King GJ (1998). Reliability of range-of-motion measurement in the elbow and forearm. J Shoulder Elb Surg.

[28] Singh HP, Dias JJ, Slijper H, Hovius S (2012). Assessment of velocity, range, and smoothness of wrist circumduction using flexible electrogoniometry. J Hand Surg Am.

[29] Yen TY, Radwin RG (2000). Comparison between using spectral analysis of electrogoniometer data and observational analysis to quantify repetitive motion and ergonomic changes in cyclical industrial work. Ergonomics.

